# Effectiveness, perceptions and environmental benefits of remote consultation for adults referred with recurrent tonsillitis

**DOI:** 10.1308/rcsann.2022.0098

**Published:** 2023-02-13

**Authors:** T Gupta, P Bowles, MF Bhutta

**Affiliations:** ^1^ University Hospitals Sussex NHS Foundation Trust, UK; ^2^ Brighton & Sussex Medical School, UK

**Keywords:** Remote consultation, Telephone, Tonsillitis, Carbon footprint, Patient satisfaction

## Abstract

**Introduction:**

We evaluate remote consultation for adult patients referred with recurrent sore throat, measuring the effectiveness of the consultation, satisfaction and environmental impact.

**Methods:**

Eligible patients were invited to telephone clinics, undertaking a satisfaction survey after consultation, focusing on perceived convenience, satisfaction, cost and travel arrangements (used to calculate potential environmental benefit). Provider opinion was also captured.

**Results:**

Forty-eight of 60 patients attended, with 38 (63%) eligible for inclusion. Thirty-six of these 38 patients (95%) had a definitive outcome of tonsillectomy (27/38) or discharge (9/38). Thirty-three of the 38 patients (87%) responded to the survey and reported high satisfaction in all arms of questioning (mean Likert ranking = 4.7/5). A mean of 3.76 hours of missed work and 5.17kg carbon dioxide emission equivalents were saved per patient. Provider responses were positive towards ongoing remote consultation use.

**Conclusions:**

Telephone consultation for adult patients considered for tonsillectomy is convenient to patients in terms of cost and time, reduces environmental harm and is associated with high patient and provider satisfaction.

## Introduction

Remote consultation offers the opportunity for greater patient convenience, improved health access and lower environmental harm (predominantly from reduced patient travel).^1,2^ In a recent review, we found that remote consultation in the ear, nose and throat (ENT) department was associated with efficient and cost-effective patient pathways and high patient satisfaction.^3^ However, between 13% and 72% of patients required a subsequent face-to-face consultation, evidencing a need to further evaluate which clinical presentations and/or patient groups are most suited to remote assessment.

Recurrent sore throat is a common presentation in primary care, reflected in a high volume of referrals to ENT specialists for consideration of tonsillectomy.^4^ Candidacy for surgery is based upon severity and frequency of episodes of sore throat,^5^ and examination findings are usually irrelevant (especially as referred patients will rarely be seen during an acute episode), making this condition potentially well suited to remote assessment. Our objectives here were to evaluate remote consultation for adults referred with symptoms of recurrent tonsillitis, to determine the effectiveness of the consultation, patient and provider perception and satisfaction, and mitigation of patient-related financial and environmental harm.

## Methods

### Ethical review

Our hospital research department confirmed that this study was exempt from formal ethical review. Data were collected and stored anonymously.

### Study population and setting

We invited 60 sequential eligible adult patients routinely referred from primary care for recurrent acute tonsillitis (previously diagnosed and managed by their general practitioner in the community) to a telephone consultation. Referral was for consideration of surgical management with patients having been assessed as having met the threshold as defined by national guidelines.^5^

Exclusion criteria included red-flag signs (e.g. dysphagia, voice change and weight loss) that could signify head and neck cancer, and also tonsillar asymmetry, symptoms of sleep-disordered breathing or other pathology necessitating examination. All participants were offered the option to not participate and convert to a face-to-face appointment (held at a later date) if preferred.

Participants were scheduled into one of five bespoke telephone clinics between March and July 2021 (during the COVID-19 pandemic), conducted by one of two consultant ENT surgeons (PB and MFB). Each appointment was allocated 15 minutes. If a participant did not answer the phone, one further attempt was made 15–30 minutes later. Effectiveness of consultation was defined as the proportion of participants receiving definitive care: either listed for tonsillectomy or discharged.

For those listed for tonsillectomy, consent was taken over the phone and recorded as a discussion of the risks vs benefits of surgery. Patients were then directed to online information on ENT-UK for optional further reading. All patients also underwent a telephonic preoperative assessment by a member of the ENT nursing team. The next contact with a surgeon was on the morning of surgery, as is our usual practice (unless the patient subsequently specifically requested an interim repeat consultation).

The lead author phoned each participant 5–10 minutes following the consultation to gather their opinions using a structured proforma (Appendix 1, available online) that was based upon existing resources.^6-9^ Questions were asked regarding convenience, communication, overall satisfaction and future use of remote consultation, using a mixture of 5-point Likert scale responses, yes/no and open response questions.

### Assessment of patient-related financial and environmental cost

Participants were asked if they were employed and if remote consultation had enabled them to take less time away from employment. They were also asked what method(s) of transport they would have used to attend if a face-to-face consultation had been scheduled, and the associated distance and cost. This was used to calculate the associated carbon footprint using UK government estimates of per kilometre and per passenger carbon dioxide emission equivalents (CO_2_e) for an average petrol car, local bus or train.^10^ Travel distances were estimated using the shortest route on Google Maps (Google Inc., Mountain View, CA, USA).

### Assessment of provider perception and satisfaction

After completion of the clinics, a proforma (Appendix 2, available online) was provided to the two ENT consultants to capture their opinion on convenience, communication, clinical assessment and future use of remote consultation. This included Likert ranking of questions from 1 (strongly disagree) to 5 (strongly agree) and open responses.

### Outcomes at surgery

Outcomes of patients listed for surgery were audited one year following the initial run of the study.

## Results

Of 60 patients invited to clinic, none withdrew from the invitation; 48 patients attended their appointment (48/60, 80%), 8 patients did not answer when phoned and 4 patients answered but were unable to participate because they were unaware of the schedule and had competing commitments. Ten patients attending their appointment were excluded from progression to the next stage of the study because six were deemed unsuitable for inclusion owing to their primary issue differing from their referral notes (including enlarged tonsils, tonsiloliths, sleep apnoea or nasal or ear symptoms), and four patients wished to pursue care in the private sector.

Of the 38 remaining patients (24 women and 14 men, age range 19–43 years, mean age 25 years), 27 were listed for tonsillectomy (27/38, 71%) and 9 (9/38, 24%) were discharged back to primary care. Thirty-six patients (95%) received definitive care at their appointment. The remaining two patients did not meet the criteria for surgery but were offered follow-up appointments. One patient listed for surgery also had a follow-up appointment to evaluate additional ear symptoms.

### Patient perception and satisfaction

Thirty-three patients (87%) responded to the survey phone call, and the results are summarised in Tables 1 and 2. Three patients experienced technical issues with their consultation that were resolved by prompt call-back by the ENT surgeon. Of note, no difference in patient satisfaction was demonstrated between those listed for surgery and those discharged, with all nine discharged patients providing scores of 4 and above to the Likert ranking questions. No patient who had been discharged felt as though care had been negatively impacted by the lack of physical examination and only one stated they would have preferred face-to-face review (compared with 5 of the 27 listed for tonsillectomy).

**Table 1:** Likert ranking questions: patient responses

Question	Likert score					Mean Likert	Proportion rating 4 or 5 (%)
	1	2	3	4	5		
This appointment is important to my health and wellbeing	0	2	6	11	14	4.1	25/33 (76)
This appointment saved me money	2	1	4	4	22	4.3	26/33 (79)
This appointment saved me time	0	0	2	5	26	4.7	31/33 (94)
Telephone consultation made receiving care more accessible	0	0	3	5	25	4.7	30/33 (91)
All my concerns were addressed	0	0	0	2	31	4.9	33/33 (100)
Enough time was given for my consultation	0	0	0	3	30	4.9	33/33 (100)
I had enough time to ask questions	0	0	0	1	32	5	33/33 (100)
Overall, I am satisfied with telephone consultation	0	0	1	3	29	4.8	32/33 (97)
I would be happy to have another telephone consultation	0	2	2	4	25	4.5	29/33 (88)

**Table 2:** Closed questions: patient responses

Question				Majority response (%)
	Yes	No	Unsure	
Was your care negatively impacted by lack of physical exam?	3	29	1	No (88)
Was anything missed because you weren’t seen in person?	0	31	2	No (94)
Would you have preferred face-to-face consultation?	6	27	0	No (82)
Would you have preferred video consultation?	6	27	0	No (82)
Do you mind discussing surgery over the phone?	1	32	0	No (97)

### Patient-related financial and environmental cost

Had their appointments been face-to-face, 16 patients would have used a car to travel to and from the hospital, 14 would have used public transport (bus or train) and 3 would have walked. Mean cost of return travel was £8.24 (after excluding those walking, unsure of cost or with bus passes) and the mean return travel time was 71.5 minutes.

Mean carbon emissions for patients (*n* = 16) making a return journey by car was 8.77kg CO_2_e (mean return distance travelled = 39.48km), for patients (*n* = 11) travelling by bus, 1.31kg CO_2_e (mean return distance of 8.86km) and for patients (*n* = 3) travelling by rail, 5.35kg CO_2_e (mean return distance of 121.2km). This equates to an overall mean of 5.17kg CO_2_e for all 33 patients.

Twenty patients (61%) reported they would have had to take time off work had their appointment been face-to-face (11 full days and 9 half days). Assuming a working day of 8 hours, this equates to 124 hours of lost work avoided, a mean of 3.76 hours for all 33 respondents. Three patients also mentioned that telephone consultation mitigated the need to organise childcare.

### Other comments

Ten patients offered general feedback, and all commented on the increased convenience of a telephone consultation as opposed to face-to-face. Several commented that during the pandemic, telephone consultation meant they did not have to wait a long time for the next in-person appointment. Many also stated that although they felt ‘apprehensive’ or ‘dubious’ prior to their appointment, afterwards they felt that unlike in-person appointments, they felt ‘heard’ or ‘listened-to’.

Two patients commented they would have preferred to have physically met a member of the operating team before being listed for surgery.

### Provider feedback

Both consulting ENT surgeons opined that a telephone consultation is cost-saving (Likert 5, 5), communication by telephone was straightforward (4, 4), and disagreed that decision-making was negatively impacted by the lack of an examination (2, 2). There was a mixed opinion on whether telephone consultations saved time (3, 5). Both were satisfied with the telephone consultation (4, 5) and would be happy to continue its use for this purpose (4, 4). Both also felt that a telephone consultation was as effective as an in-person consultation, and neither would have preferred a face-to-face consultation.

General comments included that telephone clinics worked better than expected. One provider mentioned that although face-to-face interaction with the patient is a rewarding aspect of the job, they felt for this indication, remote consultation worked well when also considering benefits of reduced cost, emissions and inconvenience to the patient.

Certain limitations were also raised. An inability to visually evaluate body mass index was an impediment to assessing anaesthetic risk. It was also apparent that several patients were simultaneously engaged in other activities, which at times impacted the flow of communication and led to a disjointed and prolonged consultation.

### Outcomes at surgery

At the point of re-auditing, 18 of the 27 patients listed for tonsillectomy had undergone surgery (67%) with 12 (33%) remaining on the waiting list (wait for surgery was prolonged due to the COVID-19 pandemic). Of those who had undergone tonsillectomy, there were no unexpected findings at surgery and all patients were discharged the same day without complication.

## Discussion

We found that in assessing patients referred for recurrent sore throat, remote consultation was effective and was associated with high patient and provider satisfaction. Patient feedback showed high satisfaction in all arms of the survey, including convenience in terms of time and money saved, and increased accessibility, irrespective of consultation outcome. Of those listed for surgery who had undergone tonsillectomy by the time of auditing, none had unexpected or concerning findings at operation. Both ENT providers were satisfied with the telephone consultation, did not feel it impacted on clinical assessment and were happy to use it again.

Our findings of high patient satisfaction with remote consultation mirror the findings of other studies. For example, in one recent prospective study comparing patient satisfaction rates between telephone and face-to-face ENT consultation using written questionnaires including Likert ranking questions, high rates of satisfaction were found, with no statistically significant difference between the two consultation modes.^11^ More specifically, studies have also demonstrated high rates of patient-perceived convenience in terms of time and money, as well as avoiding missed time from work or school and eliminating difficulties of travelling for the frail or less mobile.^12-15^

We also quantified a mean of 3.76 hours of missed work and 5.17kg CO_2_e from travel mitigated per patient. Tonsillectomy is a common procedure in the UK, with 40,103 performed in 2019–2020, of which 15,126 were in working age adults.^16^ In our cohort, 71% (27/38) of consultations ended in listing for surgery; in previous studies of in-person consultations, the comparable figure was 81% (148/184)^17^ and 85% (125/147),^18^ giving an aggregate of 81% (300/369). Extrapolating our findings, if 81% of consultations ended in surgery, then we estimate around 18,600 (15,126/0.81) adult ENT consultations for recurrent sore throat occur in the UK each year, and if all were performed remotely, this would save just under 70,000 working hours (3.76 hours × 18,600) and over 96,000kg CO_2_e from travel associated with clinic attendance (5.17 CO_2_e × 18,600).

Importantly, remote consultation in the context of recurrent sore throat was also found to be effective, with almost all consultations resulting in a definitive endpoint (surgery or discharge). When comparing our rate of listing for tonsillectomy with those seen face-to-face in the two studies mentioned above, no significant difference was found (27/38 vs 300/369, *p* = 0.1209, two-tailed Fisher’s exact test).^17,18^

We believe that remote rather than face-to-face consultation for adult patients with recurrent sore throat could, and perhaps should, become the norm. It is likely this could also be applied to older children referred with recurrent sore throats. However, and particularly with younger children, there may be concerns of sleep-disordered breathing and a loss of opportunity to perform safe-guarding checks, so face-to-face consultation and physical examination is likely still necessary.^19^

Our findings did highlight opportunities to improve the efficiency of our process. Four patients were not aware of their appointment when contacted, highlighting a need for better hospital communication, and six patients were excluded because of an inappropriate presenting complaint, which would be improved through better triage.

### Study limitations

There were limitations to our study. Our sample size was small, reducing precision in our estimates of outcomes. We did not have a control arm of face-to-face consultation, but given our findings, a control arm would be unlikely to change our conclusions in support of remote consultation. The study was conducted during the COVID-19 pandemic and patients may have had differing views outside this context. Finally, not all patients listed for tonsillectomy had their surgery more than 1 year on, meaning we had incomplete data on the final endpoint of our study.

## Conclusion

Remote telephone consultation for patients referred with recurrent sore throat for consideration of tonsillectomy has benefits to both the patient and provider in terms of cost, time and environmental harm, and is associated with high patient and provider satisfaction.

## Data Availability

Data that support the findings of this study are available in the only supplementary material.
